# Evaluation of Knowledge Among Interns in a Medical College Regarding Palliative Care in People Living with HIV/AIDS and the Impact of a Structured Intervention

**DOI:** 10.4103/0973-1075.78443

**Published:** 2011

**Authors:** Sameer Valsangkar, Trupti N Bodhare, Shripad B Pande, Samir D Bele, B Sitarama Rao

**Affiliations:** Department of Community Medicine, Prathima Institute of Medical Sciences, Nagunur, Karimnagar, Andhra Pradesh, India; 1 Department of Internal Medicine, Prathima Institute of Medical Sciences, Nagunur, Karimnagar, Andhra Pradesh, India

**Keywords:** Medical interns, Palliative care in HIV/AIDS, Structured intervention

## Abstract

**Background:**

The evolving nature of palliative care and its renewed role in people living with HIV/AIDS (PLWHA) in the post-HAART (highly active anti-retroviral therapy) era warrants an evaluation of the present curriculum in medical under graduates.

**Objectives:**

The objectives are(1) to measure the existing knowledge regarding palliative care and its application to PLWHA among medical interns and (2) to measure the impact of a structured intervention on knowledge dimensions.

**Design and Setting:**

Interventional repeated measures study.

**Materials and Methods:**

A convenience sample of 106 interns in the medical college completed a pre-test assessment and a post-test assessment following a structured intervention for evaluation and comparison of knowledge over three dimensions which were (1) knowledge of palliative care and its application in PLWHA, (2) medical symptoms in PLWHA requiring palliative care and (3) psychosocial needs in PLWHA requiring palliative care.

**Results:**

The mean scores on knowledge showed a consistent increase after the structured intervention and Student’s t-test was significant across three dimensions of knowledge of palliative care and its application (*t*=9.12, *P* value <0.001), medical symptoms in PLWHA requiring palliative care (*t*=12.72, *P* value <0.001) and psychosocial needs in PLWHA (*t*=11.14, *P* value <0.001).

**Conclusion:**

In spite of the unique challenges presented by the varying course of illness in PLWHA and the variety of needs on the medical, psychosocial and family dimensions, a structured approach and an integrated course curriculum involving principles of both primary and palliative care principles will improve the efficiency of the undergraduate medical education program and enable delivery of effective palliative care interventions and improve quality of life in PLWHA.

## INTRODUCTION

Since the case series in 1981 of Kaposi sarcoma to the adoption of highly active anti-retroviral therapy (HAART) and the transition of HIV/AIDS to a chronic manageable disease, palliative care has played a varying and evolving role from end of life care in the pre-HAART era to palliation of symptoms and toxicities of HAART era currently.[1] The distinction between curative and palliative components of treatment is blurred and the components are being applied concurrently with HIV/AIDS transitioning from being a disease with a steady decline toward hospice care to a chronic course with periodic crises and a more prolonged survival rate.[2,3] There is an increasing need for comprehensive care with symptom and drug toxicity management, psychosocial, family and care planning support and hence an emergent requirement for evaluating the role of palliative care principles and its role in clinical practice and medical education in the undergraduate curriculum in India.

WHO (World Health Organization) defines palliative care as “an approach that improves the quality of life of patients and their families facing the problem associated with life-threatening illness, through the prevention and relief of suffering by means of early identification and impeccable assessment and treatment of pain and other problems, physical, psychosocial and spiritual.”[4] The spectrum of symptoms in HIV/AIDS, the HAART therapy in itself with its associated toxicities, the stigma associated with the condition and the burden of opportunistic infections, psychosocial issues, impact on the life of the patient’s family members provides a plethora of opportunity for application of the WHO principles of palliative care and offer for the improvement in quality of life.

With an estimated disease burden of 1.8 to 3.2 million people living with HIV/AIDS in India,[5] these patients represent a large share of morbidity in the clinical practice at culmination of an undergraduate course with the burden likely to increase as mortality plateaus and survival rates are prolonged. In addition, previous studies[6,7] have shown that undergraduates are uncomfortable and ill equipped to handle end of life care and communication with family and in context to HIV with its complex disease process, unpredictable course and wide range of complications. In addition primary care practitioners have an excellent platform to empathize with the patients, involve community members, provide support to the family and these principles substantially overlap with the provision of high quality palliative care.

Studies[6,7] on palliative care have presented evidence-based recommendations for teaching palliative care principles which can be adapted to teaching in the primary care setting with traditional structured educational methods such as post-clinic rounds, conferences, case-based discussions, lectures and seminars with evaluation of clinical competency in palliative care through objective structured examinations. Integrated teaching and improvement in palliative care education can better equip a medical student to face challenges and also have benefits for PLWHA and their families.

The current study aimed at measuring existing knowledge of palliative care and its application to PLWHA in medical interns, the effect of a structured intervention on improvement of knowledge and application to make recommendations for an integrated curriculum incorporating primary and palliative care targeted to PLWHA.

## MATERIALS AND METHODS

An interventional repeated measures study was conducted and a convenience sample of 106 interns in the medical college completed a pre-test assessment and a post-test assessment following a structured intervention for evaluation and comparison of knowledge regarding palliative care needs in PLWHA.

Baseline characteristics regarding the intern’s age and sex, exposure to terminal and non-terminal PLWHA, completion of medicine and allied postings and attendance of formal training courses in palliative care if any were obtained. Knowledge was evaluated by a self-administered, 32-item semi-structured questionnaire factored into three dimensions, namely (1) knowledge regarding palliative care and its application, (2) knowledge regarding medical symptoms requiring palliative care in PLWHA and (3) knowledge regarding psychosocial needs requiring palliative care in PLWHA. The palliative care and application knowledge dimension consisted of seven items factored into three domains (objectives, application and integration of palliative care with curative care), the medical symptoms dimension consisted of 11 items factored into three domains (most common symptoms in PLWHA, pain management and side effects of HAART) and the psychosocial dimension of 14 items in five domains (delivery of palliative care, nutritional care requirements, palliative care in children, psychological components and end of life palliative care). The respondents completed the questionnaire both pre- and post-intervention and the mean score in each domain for the three dimensions was calculated. Student’s *t*-test was performed on the pre- and post-test scores in each dimension individually.

The structured intervention consisted of multiple interactive didactic sessions conducted over a period of 3 days by a team of faculty in the Department of Internal Medicine and the Department of Community Medicine. The content of the workshop was developed after the review of the existing curriculum and an assessment of the needs of the interns after a focus group discussion. The categories covered broadly included an overview of palliative care and its application to PLWHA, the shift from the divergence of palliative and curative care to an integrated continuum, the most common medical symptoms in PLWHA requiring medical care including pain and its management, fatigue and sleep disturbance, initiation of HAART and its adverse effects, delivery of palliative care, end of life care, nutritional and spiritual needs of the patient, social support, special needs in children and psychological symptoms. Post-intervention, the questionnaire was re-administered.

Statistical analysis was performed using PASW software version 18 (SPSS) and measures obtained were percentages, descriptives (mean and 95% confidence intervals), box plots for data presentation and paired tests of significance and *P*-values (Student’s *t*-test).

## RESULTS

Table 1 describes the general characteristics of the respondents including gender, age and the number of PLWHA examined by them who were non-terminal and terminal. There was an equitable distribution of gender with 51 males and 55 females and a narrow range for age from 21 years to 24 years. Interns who had attended medicine and allied posting had more exposure to examining PLWHA with 53.7% of the respondents having examined less than 11 PLWHA who were non-terminal and 77.4% having examined less than 11 PLWHA who were terminal.

**Table 1:** Characteristics of the respondents

		N	%
Gender	Male	51	48.1
	Female	55	51.9
Age	21	3	2.8
	22	47	44.3
	23	43	40.6
	24	13	12.3
Formal training in palliative care	Yes	27	25.5
	No	79	74.5
Number of patients with HIV/AIDS	None	24	22.6
examined	1–5	25	23.6
	6–10	8	7.5
	11–15	15	14.2
	16–20	22	20.8
	>20	12	11.3
Number of terminal patients with HIV/	None	45	42.5
AIDS examined	1–5	17	16
	6–10	20	18.9
	11–15	16	15.1
	16–20	4	3.8
	>20	4	3.8
Total (n)		106	100

**Figure 1:**
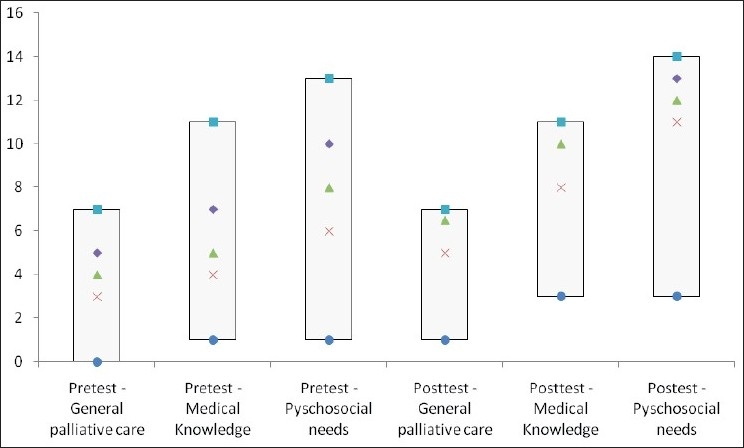
Score distribution of the respondent’s knowledge on the domains of general palliative care, medical symptoms and psycho-social needs in pre and post tests

Figure 1 shows the overall comparison of the pre- and post-test scores on the three dimensions of overall knowledge regarding palliative care, medical symptoms in PLWHA requiring palliative care and psycho-social needs of PLWHA requiring palliative care. The minimum score, 25^th^ percentile, median, 75^th^ quartile and maximum score are depicted in the box plot. The median, represented by a square block, shows a consistent rise across the three dimensions on the pre- versus post-test after intervention.

A *t*-test was performed on the test scores on the individual dimensions and the score and the *t*-values are depicted in Tables 2–4. The mean scores and the 95% CI of mean on each domain in the three dimensions are also depicted in the above tables. On the dimension of knowledge regarding palliative care, among the domains of objectives, application of palliative care and integration with curative care, increase in mean scores were obtained on all three domains with a significant *t*-test (*t* = 9.12, *P* value <0.001) on comparison with cumulative scores across pre- and post-test. Similarly, cumulative scores for the domains in the medical symptoms in PLWHA requiring palliative care dimension and psycho-social needs of PLWHA requiring palliative care dimension were obtained and the *t*-test was significant in both (*t*=12.72, 11.14 and *P* value <0.001, 0.001, respectively). The domains on the dimension of the medical symptoms in PLWHA requiring palliative care (knowledge of common symptoms in PLWHA, pain management and ART and its side effects) and psycho-social needs of PLWHA requiring palliative care (knowledge regarding delivery of palliative care, nutritional care requirements, palliative care in children, psycho-social components and end of life care) all showed a consistent increase across mean scores.

**Table 2:** Knowledge regarding palliative care and its application

Factors	Range of scores	Pre test mean scores and 95% CI	Post test mean scores and 95% CI
Objectives of palliative care	0–3	1.65 (1.43–1.87)	2.54 (2.38–2.7)
Application of palliative care	0–3	1.72 (1.53–1.91)	2.52 (2.38–2.65)
Integration of palliative care with curative care	0–1	0.45 (0.36–0.55)	0.81 (0.74–0.89)
Total	0–7	3.82 (3.49–4.15)	5.87(5.58–6.16)

*t value = 9.120, d.f. =105, *P* value <0.001

**Table 3:** Knowledge regarding medical symptoms requiring palliative care in HIV/AIDS

Common symptoms	Range of scores	Pre test mean scores and 95% CI	Post test mean scores and 95% CI
Common symptoms requiring palliative care	0–4	2.19 (1.97–2.40)	3.47 (3.31–3.63)
Pain management in HIV/ AIDS	0–3	1.58 (1.41–1.74)	2.58 (2.45–2.72)
ART and its side effects	0–4	1.90 (1.65–2.14)	3.28 (3.1–3.46)
Total	0-11	5.66 (5.21–6.11)	9.34(9–9.68)

*t value = 12.721, d.f. =105, *P* value <0.001

**Table 4:** Knowledge regarding psychosocial needs requiring palliative care in HIV/AIDS

Factors responsible	Range of scores	Pre test mean scores and 95% CI	Post test mean scores and 95% CI
Delivery of palliative care	0–4	2.42 (2.18–2.67)	3.39 (3.2–3.57)
Nutritional care requirements	0–2	0.99 (0.84–1.14)	1.61 (1.48–1.74)
Palliative care in children	0–1	0.5 (0.4–0.6)	0.77 (0.69–0.85)
Psychological components of palliative care	0–4	2.25 (2–2.51)	3.34(3.16–3.52)
End of life palliative care	0–3	1.59 (1.4–1.78)	2.58(2.44–2.71)
Total	0–14	7.76 (7.23–8.3)	11.69(11.25–12.13)

*t value = 11.148, d.f. =105, *P* value <0.001

## DISCUSSION

Palliative care training in the undergraduate medical curriculum is loosely structured and ill defined in countries like USA[6,7] and India with striking inadequacies and stop gap arrangements especially in the clinical years. With a shift from a propensity to infectious diseases to chronic life style diseases, morbidity care takes precedence over mortality for a general practitioner following a course in undergraduate medical education. HIV/AIDS presents a unique clinical course being both infectious and requiring chronic care over the years which presents unique challenges while managing the palliative aspects of the disease. To identify, evaluate and improve curricular offerings and teaching formats for the best care and improvement of quality of life for the patient and to provide a well-rounded clinical training for medical undergraduates is an essential requirement.

A similar study by Velayudhan *et al*[8] introduced palliative care training among medical students and nurses and measured the impact of educational intervention through five sessions of didactic lectures and measured knowledge through a 20-item pre- and post-test questionnaire. The primary focus of their intervention was palliative symptom relief and the study concluded that patient-centered communication, ethical issues, decision making at the end of life, whole person care and interdisciplinary work integrated in the entire curriculum can have a lasting impact on future medical practice. Emphasis was also laid on the dimensions of palliative care to be covered including pain, symptom assessment and treatment, ethical and legal aspects in end of life care, communication skills, personal reflection, and psychosocial, spiritual and cultural aspects of death, interdisciplinary team efforts and the possible setting for palliative care as covered in the present study.

Palliative care needs in PLWHA have been evaluated in a study by Uwimana *et al*,[9] in a quanti-qualitative study in health care workers and PLWHA and more than half of health care professionals reported an absence or lack of palliative care training. The major unmet needs with respect to palliative care in PLWHA included pain relief, symptom management, financial assistance and nutritional support. Accessible health care for PLWHA through efforts to train health care workers in palliative care and alteration in policy making were suggested as core measures to improve quality of life in PLWHA which again reflects upon the essentiality of this study.

Easterbrook *et al*[3] described the changing epidemiology of HIV infection and new challenges for HIV palliative care with the major areas covered being symptomatic and supportive care for all stages of HIV, use of HAART along with prevention and management of opportunistic infections, management of drug toxicities, treatment failure and end of life care, fluctuation in prognosis and difficulty in identifying the terminal phase, drug interactions, isolation, stigma and lack of social support and hospice versus home care. Knowledge regarding these and other issues deemed necessary in the focus group discussion by the interns was evaluated pre- and post-intervention and discussed during the intervention and resulted in an uniform increase in performance in the post-intervention test.

Billings *et al*,[6] conducted a meta-analysis on the status of palliative care education and assessed the methodology and quality of the educational program, process of evaluation of knowledge obtained and conclusions across citations of surveys on Medline in palliative care journals. Integration of palliative care training with primary care training and emphasis on palliative care training in the undergraduate medical curriculum in studies by Billing *et al*,[6] and Block *et al*,[7] provide further directions and suggestions for improving core competencies, helping medical interns understand and apply these principles in management of needs of PLWHA and evaluation of their application through objective examination of skills.

The trajectory of AIDS illness and the benefits of application of palliative care intervention at an early onset in disease progression to reduce the burden of AIDS epidemic were the focus of the study by Grady *et al*.[10] The management and the fallible approach of applying hospice care in cancer patients to PLWHA instead of targeted ways to approach and provide care in the unique circumstances of PLWHA during the end of life care for AIDS have also been evaluated in the same study.

In culmination, applying the principles, knowledge and recommendations gleaned through these studies and application of the intervention, initiatives and evaluation methods helped develop a rounded approach to the introduction of palliative care principles in medical interns targeted to PLWHA and the significant test results after comparison of pre- and post-intervention scores in the present study provides a framework for further development of a palliative care curriculum which is both beneficial to the medical interns and patients receiving their care.

## CONCLUSION

In spite of the unique challenges presented by the varying course of illness in PLWHA and the variety of needs on the medical, psychosocial and family dimensions, a structured approach and an integrated course curriculum involving principles of both primary and palliative care principles will improve the efficiency of the undergraduate medical education program and enable delivery of effective palliative care interventions and improve quality of life in PLWHA.

## Limitations

The intervention and knowledge examination relied largely upon classroom methods of teaching and evaluation which form the core of the examination-oriented approach in medical education in India. A skill-based competency approach with involvement of simulated or real PLWHA and case-based studies will further help orient the approach toward an effective development of curriculum in future evaluations.
